# Rising to the challenge

**DOI:** 10.1098/rsob.200412

**Published:** 2021-01-20

**Authors:** Jon Pines

**Affiliations:** Cancer Biology, Institute of Cancer Research, London SW7 3RP, UK

Open Biology will begin its 10th year in 2021, and this will be accompanied by a number of exciting new initiatives. Some of these originated with new members of the editorial board, while others have been in response to external events. Like many of you, I am writing this from my impromptu office in our spare bedroom as we prepare for a constrained Christmas and New Year. 2020 has proved a challenging year, but one that dramatically illustrates the importance of the unconstrained and rapid exchange of ideas. The unprecedented speed with which SARS-CoV-2 was isolated, sequenced and, just as importantly, published online, enabled the roll out of effective vaccines less than one year later. The free exchange of ideas was one of the principles on which The Royal Society was founded 360 years ago, and the guiding principle behind the founding of Open Biology as the first open-access journal of the Royal Society just a little later, in 2011.

As the pandemic unfolded, preprints reporting on new viral variants, on the routes of infection, and on the replication of the virus, were influential in the speed with which potential therapeutic avenues were identified and validated. Recognizing their role, we appointed Michael Ginger, University of Huddersfield, as Preprint Editor for Open Biology, who has quickly assembled a highly motivated and enthusiastic team of volunteers to assist him (https://royalsociety.org/blog/2020/07/introducing-open-biology-preprint-team/). Open Biology is thus very well positioned to take advantage of the increased importance being given to preprints and the open-access publishing model.

2020 brought another unwelcome challenge in the requirement for an editorial retraction of two papers that were generated by a ‘paper mill' using cloned data. The events of 2020 have shown how important it is to have credible and trustworthy sources of evidence, and this example of ‘fake news' prompted us to set in place standards for all data submitted to Open Biology and instigate image integrity and plagiarism monitoring for all papers accepted for publication [1].

2020 for Open Biology began with David Glover handing over the baton as Editor in Chief to me, and I am immensely grateful to David both for establishing Open Biology on firm foundations, and for continuing to build the reputation of the journal through commissioning high-quality reviews in his role as Reviews Editor. Of the more than 100 reviews that David has commissioned, 71 have been published thus far. I am also indebted to Buchi Okereafor and Phil Hurst at Royal Society Publishing for their patient guidance in my new role.

Sadly, we have said farewell to a number of associate editors this year—Tim Clausen, Enrico Coen, Peter Cresswell, Yukiko Goda, Stephan Grill, Sebastian Jessberger, Elena Levashina, Hong Ma, Oscar Marin, Matt Neale, Ben Nichols and Maddy Parsons—and I would like to thank them for their invaluable service. With their help and support, and that of our other editors and associate editors, in the last year we have seen a 24% increase in the overall number of submissions, including a 50% increase in submissions from the UK and the USA. This increased recognition of Open Biology is reflected in an increase in the average number of citations for Open Biology papers (Impact Factor 4.93, https://royalsocietypublishing.org/rsob/citation-metrics).

This year, we have welcomed two new associate editors, Alessandro Vannini (Human Technopole, Milan) and Julie Welburn (Edinburgh University), who strengthen the structural biology expertize of the journal, and four new Editors, Anne Bertolotti (MRC LMB), Iain Hagan (CR UK Manchester Cancer Institute), Natalie Strynadka (University of British Columbia) and Faith Osier (Heidelberg University) (https://royalsociety.org/blog/2020/03/open-biology-welcomes-new-editors-to-the-board/).

It is thanks to Anne and Iain that we have introduced a new section to Open Biology papers—‘Opening Up'—where authors can put their results in wider context; our hope is that this will foster increased discussion and debate and make Open Biology a forum for the robust exchange of ideas [2]. Complementing this are the ‘Open Questions’ articles introduced by Martha Cyert (Stanford University) [3], where researchers can outline key unanswered questions or identify neglected but important areas of study; and Special Features to provide in-depth analysis of particular areas, the first of which will be on the dynamics of protein fatty acylation (Guest Edited by Marilyn Resh, Memorial Sloan Kettering Cancer Center). We aim to go further still in 2021 by piloting moderated online debates to examine major issues, and by introducing tools to enable readers to comment upon articles online.

Thus, despite the trials of 2020, I believe that Open Biology is in a strong position—not just to meet the challenges of the next 10 years but to lead the scientific exchange of ideas that will be crucial to overcome them.
